# Towards the Development of a Substance Abuse Index (SEI) through Informatics

**DOI:** 10.3390/healthcare9111596

**Published:** 2021-11-20

**Authors:** Nikhila Guttha, Zhuqi Miao, Rittika Shamsuddin

**Affiliations:** 1 Department of Computer Science, Oklahoma State University, Stillwater, OK 74078, USA; nikhila.guttha@okstate.edu; 2 Center for Health Systems Innovation, Oklahoma State University, Stillwater, OK 74078, USA; zhuqi.miao@okstate.edu

**Keywords:** substance abuse, medicinal drug dependence, substance effect index, machine learning, standard measure, logistic regression

## Abstract

Substance abuse or drug dependence is a prevalent phenomenon, and is on the rise in United States. Important contributing factors for the prevalence are the addictive nature of certain medicinal/prescriptive drugs, individual dispositions (biological, physiological, and psychological), and other external influences (e.g., pharmaceutical advertising campaigns). However, currently there is no comprehensive computational or machine learning framework that allows systematic studies of substance abuse and its factors with majority of the works using subjective surveys questionnaires and focusing on classification techniques. Lacking standardized methods and/or measures to prescribe medication and to study substance abuse makes it difficult to advance through collective research efforts. Thus, in this paper, we propose to test the feasibility of developing a, objective substance effect index, SEI, that can measure the tendency of an individual towards substance abuse. To that end, we combine the benefits of Electronics Medical Records (EMR) with machine learning technology by defining SEI as a function of EMR data and using logistics regression to obtain a closed form expression for SEI. We conduct various evaluations to validate the proposed model, and the results show that further work towards the development of SEI will not only provide researchers with standard computational measure for substance abuse, but may also allow them to study certain attribute interactions to gain further insights into substance abuse tendencies.

## 1. Introduction

Some medicinal drugs/substances, such as opioids, have the ability to prominently affect the human nervous system by acting on chemical receptors distributed widely throughout the human body, including the brain and spinal cord. This ability is a double-edged sword; while carefully regulated use of these substances can potentially address major health issues such as chronic pain, and chemotherapy induced nausea/vomiting, they can also cause substance dependence in patients even when used for medicinal purposes. In general, medicinal drugs that exert their influence on the human body by modifying the signal pathways in the reward center of the brain [1,2,3] can become addictive and easy to misuse. As a matter of fact, drug dependence has been pervasive and an increasing concern in United States. According to Centers for Disease Control and Prevention, National Center for Health Statistics, there were 70,237 drug overdose deaths in 2017, a 12.9 times increase since 2007. Almost half of these (approximately 47,600) were due to opioid prescription drugs. Drug dependence often leads to deaths, psychosis, and much more through overdosing, abuse, and/or the patient’s inability to mitigate damaging behavior.

Various factors can increase an individual’s risk of developing a dependency, making some more susceptible than others. After the opioid crisis, various preventive measures to address opioid overdose have been implemented at federal, state and local government level. They are working with researchers, and health professionals to raise public awareness, improve access to treatment, improve medication, and much more. However, there are a variety of other medicinal drugs that pose similar risks. Examples include marijuana, benzodiazepines, phentermine, amphetamine, etc. Yet there is currently very little computational support for conducting comprehensive studies that can quantify the effect of these medicinal drugs on individual level, when prescribed separately or together. This is largely due to the fact that not everyone reacts the same way to these drugs, which often results in inconclusive scientific cohort studies. Moreover, drugs, such as marijuana that only recently got legalized, are already accessible to public even though researchers are still in the process of conducting conclusive scientific studies. Thus, it is highly likely that by the time FDA-approved cannabinoid medications are available, some people will already be pre-disposed to substance dependency and abuse. To complicate matters, despite the prescriber’s guideline, there will always be external forces pushing for increased prescription of certain drugs.

Two papers that address similar issues are presented in [4,5]. The paper [5] uses Neural Networks, Random Forests, Support Vector Machines, and feature importance to differentiate between addictive and non-addictive persons using a socio-economic survey dataset that samples a population in Dhaka, Bangladesh. On the other hand, ref. [4] uses Cerner EMR dataset to predict encounter level substance abuse cases using deep networks. While both provides interesting insights and demonstrates the use of cutting edge computational technology, neither explores the possibility of developing a patient level metric that is proportional to the individual’s tendency to abuse substance. Also, existing assessments [6] of drug abuse propensity and severity are often based on the psychometric score analysis from survey questionnaires [7,8,9,10,11,12], and tested on cohorts of few hundreds of patients within a limited geographical area. Another exemplary work in the field include the use of textual information in social media networks.The paper [13] uses data from Twitter, Facebook and other websites to assess risks (measure by the F1 score of machine learning models) of abuse based on four bio-markers: HIV, amphetamines, methamphetamines, and tetrahydrocannabinol (THC, a cannabis constituent). While these are all worthy contributions to understanding and managing substance abuse, ref. [14] identifies various aspects that can be improved to further the applications of computational techniques, including machine learning. One of these aspects is standardization of data and methods.

One possible solution to these issues is to quantify the effect of addictive medicinal drugs on the individuals. Quantification of substance effect entails the development of an index, similar to those described in [6], using objective data. Throughout scientific literature, there are many studies that use some type of “index” as a standardized measure of some scientific phenomena. For example- the body-mass index [15], a measure of body fat, is often used in studying patient health.The authors of paper [16] develop wearable biosensor, which when coupled with certain types of fabrics can be used for healthcare and biomedical monitoring, and uses the thermal index to derive the relationship between time and sensitivity of the fibre-based temperature biosensor. The paper [17] proposes a rural index as a solution for developing a unified healthcare plan to address the diverse healthcare needs in rural Australia. Moreover, ref. [18] uses features obtained through the use of AI techniques to define a melanoma index, which in return can help to classify skin-lesion images as benign or malignant.

Thus, with the availability of Electronic Medical Record (EMR) data, it might now be possible to data mine and use biological data for quantifying substance effect. Hence in this paper, we investigate and test the feasibility of a **Substance Effect Index** (or **SEI** in short) using thousands of clinical data points from all across United States. An objective biologically-based metric, such as SEI, will aid in pushing forward the substance abuse related research and diagnosis through the use of informatics.

**Challenges with quantification using EMR data:** Computation to study substance abuse, through analysis of EMR data using machine learning, usually begins by labeling each encounter in the dataset as indicating addiction/substance abuse (label 1) or not (label 0). To accomplish labeling these encounters, some studies use diagnostics codes [19,20] from the Diagnostic table, while others identify and use opiates and/or opioid related drugs, such as naloxone, oxycodone, etc., from the Medication table [4]. This is a scientifically valid approach to label data because these codes in the EMR dataset are designated by healthcare experts. Regardless of whether the studies use diagnostics codes, prescribed drugs or both, the encounters are decisively labeled as “class 1” e.g., indicating substance abuse/addiction (or not). While this kind of *hard labeling* is useful to teach a machine to learn features that are associated with substance abuse/overdose, it does not teach the machine to predict the propensity or tendency of individuals towards substance abuse/overdose, because hard labeling does not account for:

i The number of times a patient requires a hospital visit due to substance abuse/overdose/addiction.
ii The differing potency of drugs e.g., carfentanil (used as an analgesic agent) is hundred times more potent than fentanyl, which in return is a order of magnitude more potent than morphine or codeine.
iii Individual differences due to genetics/biology/physiology/psychology.
IV Interaction between different hospital visits and other possible comorbidities.

**Figure 1:**
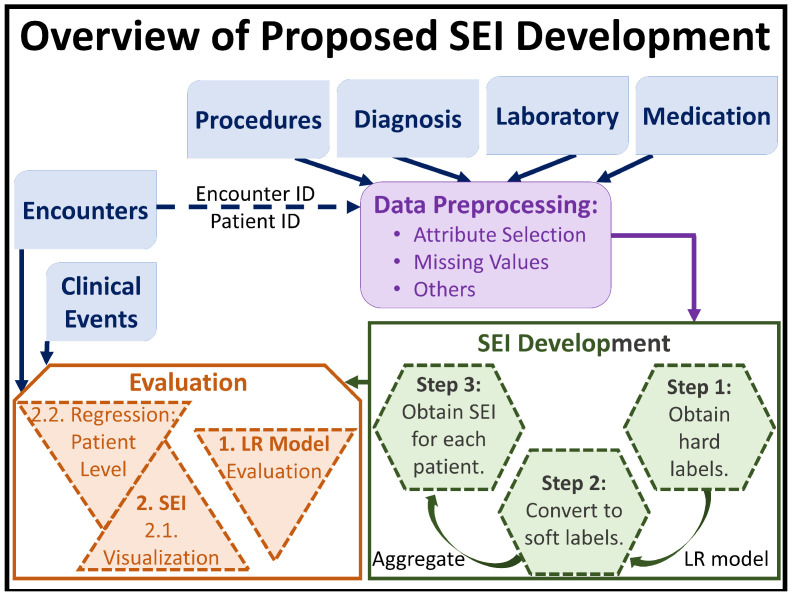
Overview of the proposed methodology and evaluation steps undertaken in this paper.

Any candidate of SEI should address the majority of the factors mentioned above. Thus, in this paper, we propose SEI (which should be a patient-level, objective measure that can quantify the effect of addictive drugs) to be a function of the EMR dataset, and use logistic regression (an existing machine learning algorithm) to find a mathematical expression for SEI. The objective nature of SEI is inevitable, because it’s definition would rely completely on clinical and biological EMR data of patients, eliminating the subjective input nature of surveys or other external influences. To best of our knowledge, even though logistic regression has been used in substance abuse studies [21], it has not been used to develop a metric or an index for substance abuse. Moreover, through the use of the mathematical expression for SEI, we are able to infer that obtaining SEI is not limited to logistic regression alone; this is discussed in more details later in this paper. Also, the use of an existing computational model for defining SEI, allowed us to relate the appropriateness of SEI directly to the performance of logistic regression model. We further validate SEI appropriateness through visual aids and analysis of patient level information retention using regression analysis. Our proposed workflow is shown in Figure 1. Once fully developed, the SEI can: (i) be used in tandem with the psychometric scores [7,8,9,10,11,12], and together they can provide a better, a more informative profile of a substance abuse patient to the clinician in-charge, and (ii) allow research advancement in the study of substance abuse by encouraging the use of standardized methods/measures. Our results are promising.

This paper is organized as follows: Section 2 gives an overview of logistic regression and the equations we use to derive a closed form expression for SEI, followed by notations, and descriptions of the datasets and pre-processing steps, while Section 2.4 delineates our proposed methodology. Section 3 provides the results we obtained from each step described in Section 2.4, and this is followed by Section 4, which discusses our observations, future work and limitations. Finally, we end with a summary of our work in Section 5.

## 2. Materials and Methods

Figure 1 gives a brief outline for the proposed process of developing SEI, on which we have been working on since Spring 2020. After prepossessing the EMR data from particular SQL tables, and obtaining the hard labels for each patient encounter using information from the dataset, we use a logistic regression (LR) model to re-calibrate the hard labels into soft labels, which are then aggregated to finally assign a scalar SEI value to a patient. As such, it is important to evaluate the LR model’s performance. Note that the Encounters and Clinical Events tables from the EMR dataset is used only during the evaluation phase of SEI. We also verify SEI via visual aids and regression analysis.

### 2.1. Overview of Logistic Regression

Even though logistic regression (LR) [22] is widely used as a binary classification model, it is by definition equipped to (i) model the probability of an event occurring given a linear combination of independent variables, (ii) estimate the *odds* of an event occurring, and (iii) predict or explain the effect of the independent variables on a binary response variable. Thus, the output of LR is a probability, *p*, of an event happening given a linear combination of independent variables. *p* is then turned into a binary response variable, yϵ[0,1] through the use of a threshold, th, such that p>th→1 and p≤th→0 (Equation (4)). The most common value for th is 0.5, and LR parameters are optimized using maximum likelihood estimate (MLE) [22].

To provide a more detailed description of LR, we use the Bernoulli variable notion of using *p* to denote the probability of an event happening, and 1−p to denote the probability of the same event not happening. The LR model then defines the odds and logit function as given in Equation (1).

(1) $$odds=\frac{p}{1-p}\;,\;\;\;and\;\;\;logit\left(p\right)=\ln\left(odds\right)=\ln\left(\frac{p}{1-p}\right)$$

Denoting the independent variables as $X_{j}$ with values $x_{j}$ respectively, and their corresponding coefficients as $\beta_{j}$, where 0<j<N and $X_{0}=1$, we obtain the following LR and estimated LR equations (Equation (3)) by setting the logit function to be equal to the linear combination of independent variables (Equation (2)):

(2) $$logit\left(p\right)=\ln\left(\frac{p}{1-p}\right)=\sum_{j=1}^{N}\beta_{j}X_{j}\Rightarrow \frac{p}{1-p}=\exp\left(\sum_{j=1}^{N}\beta_{j}X_{j}\right)$$

(3) $$p=\frac{\exp\left(\sum_{j=1}^{N}\beta_{j}X_{j}\right)}{1+\exp\left(\sum_{j=1}^{N}\beta_{j}X_{j}\right)}\;\;\;and\;\;\;\hat{p}=\frac{\exp\left(\sum_{j=1}^{N}\beta_{j}x_{j}\right)}{1+\exp\left(\sum_{j=1}^{N}\beta_{j}x_{j}\right)}$$

The final classification or binary response variable value is obtained by:

(4) $$y=\left\{\begin{array}{cc} 1 & \mathrm{if}\;\hat{p}>th\\ 0 & \mathrm{otherwise} \end{array}\right\}$$

### 2.2. Patient Notations

In this paper, an unique patient in the EMR dataset is denoted by $P_{k}$, where *k* is the patient identifier. Each $P_{k}$ can have multiple encounters, which are denoted by a time ordered set, ${}^{k}E=\left\{E_{k1},...,E_{ki},..,E_{kM}\right\}$, where $E_{k1}$ is the *encounter 1* for $P_{k}$, *i* is the index for a patient’s time-ordered encounters, and 0<i≤M. Note, using the independent variables notation introduced in Section 2.1, we can re-write encounter $E_{ki}$ using one dimensional vector form as follows: $E_{ki}=\left[{}^{ki}x_{1},...{,}^{ki}x_{j},...{,}^{ki}x_{N}\right]$.

### 2.3. Dataset

#### 2.3.1. Source and Permission

The EMR dataset used here is a subset of the healthcare database, donated to Oklahoma State University (OSU) by Cerner Corporation (https://www.cerner.com, accessed on 12 May 2021). It has clinical information on more than 60 million patients across United States, over a period of 18 years. These donated dataset is HIPAA regulated and maintained by OSU Center of Health Systems Innovations. Since the Cerner EMR databse (e.g., Health Facts©) has been completely de-identified according to HIPAA regulations, the Institutional Review Boards (IRB) at OSU exempted the study from review.

#### 2.3.2. Data Extraction

For the purpose of this paper, we extracted data of patients with ICD-10 diagnostic code starting with F11 or F12, and ICD-9 diagnostic code starting with 304 and 305. This extracted dataset has over 11 thousand unique patients with a total of over 4 million patient encounters. During at least 73 thousand of these encounters, patients were prescribed addictive medicinal drug, and over 43 thousand had drug screen test assignments. The dataset contains data from as early as September 1915 and as recent as September 2020; however, majority of the encounters are from March 2003 to June 2016. Table 1 shows a brief summary of all the relational tables present in the extracted dataset; whereas, Table 2 shows only those used in SEI developmental process, along with their relevant attributes. ${}^{k}E$ for each $P_{k}$ is created using attributes from Diagnosis, Laboratory, Procedures and Medication tables (along with patient Id and encounter Id from the Encounter table for joining the other tables).

#### 2.3.3. Preprocessing Dataset

##### Attribute Selection

We worked with the health data specialist and the health data science program manager from OSU Center for Health Systems Innovation to choose the healthcare attributes from Diagnosis, Laboratory, Procedures and Medication tables as shown in Table 2.

The Diagnosis table provides information about the diagnosis code that describes the associated diseases for the visit and its symptoms. ICD-9 codes required further processing for simplification, easy access and compatibility with ICD-10 codes.

The Laboratory table provides information about specific laboratory tests performed on a patient. Each laboratory test has a specific procedure id, description, date and time when the lab test is performed and the results of those laboratory tests, each of which records a patient’s medical history.

The Procedure table has information about the procedure Id, it’s specific code, the procedure description, and when the procedure was performed.

The Medication table records the medical drugs that are prescribed to a patient during each of their visits, the brand/generic name of the prescribed medicine, the dosage amount of the medicine, when and why the medicine was prescribed, and when the patient stopped using the medicine (if applicable).

##### Missing Values

Before obtaining Table 2, we had to handle missing values. Missing values can affect data analysis, and reduce the robustness of the results. However, filling in missing values using imputation methods can introduce bias or change the distribution of a variable. Thus, to prevent any one imputation method from having a huge impact on the data, we used a combination of imputation techniques. The Laboratory table had the highest amount of missing values and any encounter with missing values at 50% or greater was removed. Across all tables, any attribute with missing values at 70% or greater was also removed. For some attributes, missing values were filled in from related attributes (e.g., time recorded in minutes versus time recorded in seconds). The remaining missing values were filled in through other statistical imputations [23], such as cold deck imputation and mean substitution.

##### Others

Repeated Information: Repeated information (especially in the Procedure table) was combined through the use of a hash map, which aggregated repeated features among a numerical representation in one column and text description in another column.

Data Type Conversion: All the categorical, object, string, date and time data type were converted into numerically ordinal, nominal data, or statistical dummy variables using the built-in encoder functionality in Python. Numerical variables were only rescaled when necessary to keep the range of variables reasonable.

Null Values: The final step was to convert the null values to zero, because in the previous pre-processing steps we ensured that none of the attribute variables had within their range.

### 2.4. Our Proposed Method

As mentioned earlier, the purpose of this paper is to define and develop the Substance Effect Index (SEI). We expect SEI to be a *scalar*, *numerical* measure that can summarize the effect that different medical drugs, medication, etc. has on the *tendency* of *individual patients* to engage in substance abuse. Given these requirements for SEI, we divide the development of the index into 3 different steps, with stages 2 through 3 being our main contributions in this paper. In this section, we describe these 3 steps along with our rationale for each step.

*Step 1: Obtain hard labels.* SEI is a measure for an individual. However the EMR data system, which is one of the most common and highly informative clinical data system, records patient data as a series of encounters. Thus, the first step in the development of SEI is to label each encounter, $E_{ki}$ (see Section 2.2), with *hard labels* of 0 or 1 using only the DiagnosisCode and DiagnosisDescription variables from the Diagnosis relational table.
  In the EMR dataset, if an encounter was categorized as substance abuse/addiction using the ICD9 and/or ICD10 codes, and/or if the diagnosis description of the encounter contained words related to substance abuse, such as “overdose”, “abuse”, “opioid”, etc., the encounter was labeled with, and 0 otherwise. Extending the notations presented in Section 2.2, and after obtaining the hard labels, we can express the resulting encounter set of $P_{k}$ as:
  $${}^{k}Eb=\left\{\left(E_{k1},{\;}^{k}b_{1}\right),...,\left(E_{ki},{\;}^{k}b_{i}\right),..,\left(E_{kM},{\;}^{k}b_{M}\right)\right\},$$
  where ${}^{k}b_{i}$ is the hard label for $P_{k}$’s $i^{th}$ encounter, and ${}^{k}b_{i}\;\epsilon \;\left[0,1\right]$. Note, the hard labels are assigned to each encounter, and at this stage describing one individual using the hard labels would require an array/collection of hard labels.
*Step 2: Convert hard labels to soft labels.* We define SEI to be numerical because the spectrum of substance abuse patient is very wide, and defining SEI to be categorical with a limited set of values/levels would be restrictive. The wide range of the substance abuse spectrum can be attributed to individual, background, medical, and prescription strength differences. Thus, this step consists of transforming the hard labels, ${}^{k}b_{i}$, to *soft labels*.
  This transformation is achieved through the use of LR. Once ${}^{k}Eb$ has been obtained for all values of *k* (e.g., for all patients) from our previous stage, we train a LR model on the training set of encounters using ${}^{k}b_{i}$ as the response variable; in other words, we train the LR model on $E_{ki}$ for all *i* and *k* in the training set to learn/predict binary response variable.
*Step 2.1: Obtaining the soft labels.* Once we have a trained LR model, we use Equation (3) to obtain $\hat{{}^{k}p_{i}}$ for all encounters and all patients (from both training and testing sets) e.g., for all *i* and *k* in the entire dataset. This $\hat{{}^{k}p_{i}}$ now becomes the soft label for $E_{ki}$. Thus, we update the encounter set of $P_{k}$ as follows:
  $${}^{k}Ebp=\left\{\left(E_{k1},{\;}^{k}b_{1},\hat{{}^{k}p_{1}}\right),...,\left(E_{ki},{\;}^{k}b_{i},\hat{{}^{k}p_{i}}\right),..,\left(E_{kM},{\;}^{k}b_{M},\hat{{}^{k}p_{M}}\right)\right\},$$
  where ${}^{k}{\hat{p}}_{i}$ is the soft label for $P_{k}$’s $i^{th}$ encounter, and $0\leq {\;}^{k}{\hat{p}}_{i}\;\leq 1$. At the end of this stage, one individual still requires an array/collection of soft labels to be properly described.
*Step 2.2: Fine tuning the value of th.* In order to ensure that the hard labels were properly transformed into representative soft labels, we need to make sure that the LR model was properly trained. However, before calculating the evaluation metrics, we need to fine tune the value of th, because scaling the effect of the independent variables may not be cleanly encapsulated by the general threshold value of 0.5. We reason that if the model learnt properly, then soft labels ≤ 0.4 will correspond to hard coded label of 0, and soft labels ≥ 0.6 with hard label of 1. As such, we use a Receiver Operating Characteristic (ROC) curve to determine the cutoff point, cp, within the (0.4, 0.6) open interval, and accordingly set th=cp.
*Step 2.3: Evaluating the trained LR model.* Once the value of th has been determined, we use Equation (4) only on the test set to evaluate the trained LR model based on accuracy, specificity, and sensitivity.
*Step 3: SEI Definition.* We need to transform the array of soft labels for an individual patient to a scalar value. Thus, we define the SEI of $P_{k}$ as the average of soft labels found in ${}^{k}Ebp$.
  (5) $$SEI{(}^{k}Ebp)=\frac{\sum_{i=1}^{M}\hat{{}^{k}p_{i}}}{M}$$
  Finally, substituting Equation (3) within Equation (5), gives us a more comprehensive way to express SEI in terms of EMR dataset, where $\beta_{i}^{*}$ are coefficients of independent variables optimized by the LR model:
  (6) $$SEI{(}^{k}E)=\frac{\sum_{i=1}^{M}\left[\frac{\exp\left(\sum_{j=1}^{N}\beta_{j}^{*}{\;}^{ki}x_{j}\right)}{1+\exp\left(\sum_{j=1}^{N}\beta_{j}^{*}{\;}^{ki}x_{j}\right)}\right]}{M}$$

## 3. Results

The most direct method for evaluating SEI would be to ask a group of clinical physician to separately rank returning patients in order of most (or least) likely to abuse substance, and then compare their ranking to the corresponding SEI values assigned (by ML the model) to those patients. However, this is quite challenging due to time constraints on part of the clinicians; and while we do plan to take SEI to clinical trial once it is fully developed by working in tandem with our clinical collaborators, at this stage computational evaluation should suffice to demonstrate the feasibility of developing SEI. Thus for this paper, we employ the following evaluation techniques for each step described in Section 2.4.

### 3.1. Evaluation for Step 1 (Obtain Hard Labels)

Since this step involves converting textual variables into corresponding categorical variables, and since the values for textual variables are derived from healthcare expert in the field, this step requires no further evaluation.

### 3.2. Evaluation for Step 2 (Convert Hard Labels to Soft Labels)

**Figure 2:**
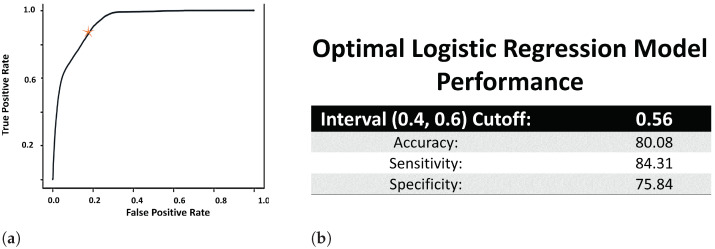
Results of the developmental process as described in Steps 2.2 and 2.3 in Section 2.4, and thus this figure displays the performance of the LR model. The star in 2a represents the model whose performance is presented in 2b. (**a**) ROC curve of LR. (**b**) Evaluation of the trained LR model, with th=0.56.

**Figure 3:**
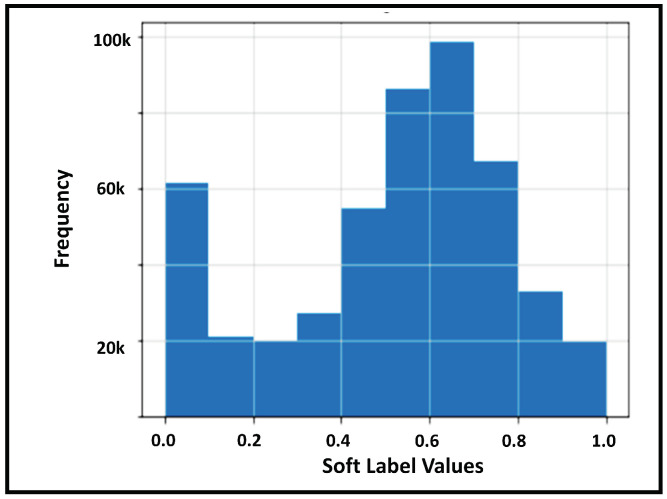
Histogram and range of SEI.

This is the crucial step in the defining SEI, where we convert the hard labels to soft labels. Thus, to test the quality of the soft labels, we evaluate the LR model using a 80-20 training-testing split. Since the LR model played the role of the converter that transformed hard labels to soft labels, the performance of the trained LR model is directly proportional to the quality of it’s output e.g., the soft labels. And as described in Steps 2.2 and 2.3 in Section 2.4:

- The ROC curve of the LR model is used to determine the cutoff point (or the value of th defined in Equation (4)) in the open (0.4, 0.6) interval. Thus, we use the area under the ROC curve (AUROC) to determine the performance of the LR model. Figure 2a displays the ROC curve of the trained LR model, which was used to set the value of th at 0.56. The AUROC value (Figure 2b) was obtained using Riemann sum method of finding the area under the curve and found to be 95% (where the underestimated AUROC was 92%, and the overestimated AUROC was 97.76%).
- The statistical significance of all the attributes in Table 2 using the LR model came out with *p*-values of 0.00*, where ’*’ indicates that the minimum *p*-value was even lower.
- Since the output of LR is a probability, *p*, LR is usually used as a classification model through the use of th as shown in Equation (4). Thus, using th=0.56 (from the ROC curve computation mentioned above), the accuracy, sensitivity and specificity of the trained LR model is calculated (and presented in Figure 2b) using the following definitions, where TP = true positive, FP = false positive, TN = true negative and FN = false negative:
  (7) Specificity=TN/(TN+FP)
  (8) Sensitivity=TP/(TP+FN)
  (9) Accuracy=(TP+TN)/(TP+TN+FP+FN)
- In addition, we also provide a histogram of soft label values obtained from the trained LR model in Figure 3. The histogram shows us that a large proportion of the encounters obtained the soft label in the proximity of values 0.4 and 0.6. Figure 3 also ensures that the range of value of the soft labels fall within the range of 0 and 1. This is to be expected because the output of the LR model, $\hat{{}^{k}p_{i}}$, is a probability, which we are regarding as soft labels. And since SEI for a patient is defined as the mean of their associated soft labels, this means that SEI is also restricted within the closed [0, 1] range.

### 3.3. Evaluation for Step 3 (SEI Definition)

Each patient, $P_{k}$ is described by a set of encounters, ${}^{k}Ebp$, where each encounter, $E_{ki}$, is assigned a hard (${}^{k}b_{i}$) and a soft label (${}^{k}b_{i}$). A scalar SEI for each $P_{k}$ is then obtained by applying Equation (6). Thus, in order to validate SEI, we take a two pronged approach: (i) Visual Validation, and (ii) Patient Level Information Retention.

#### 3.3.1. Visual Validation

**Figure 4:**
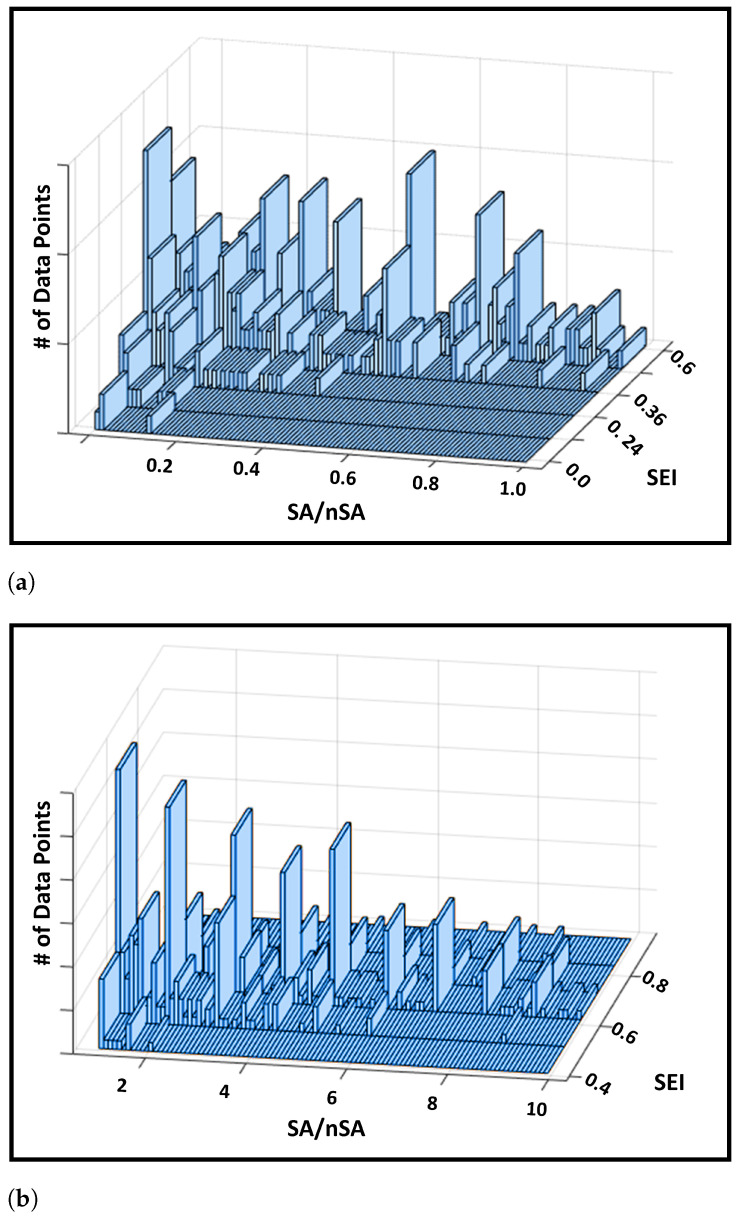

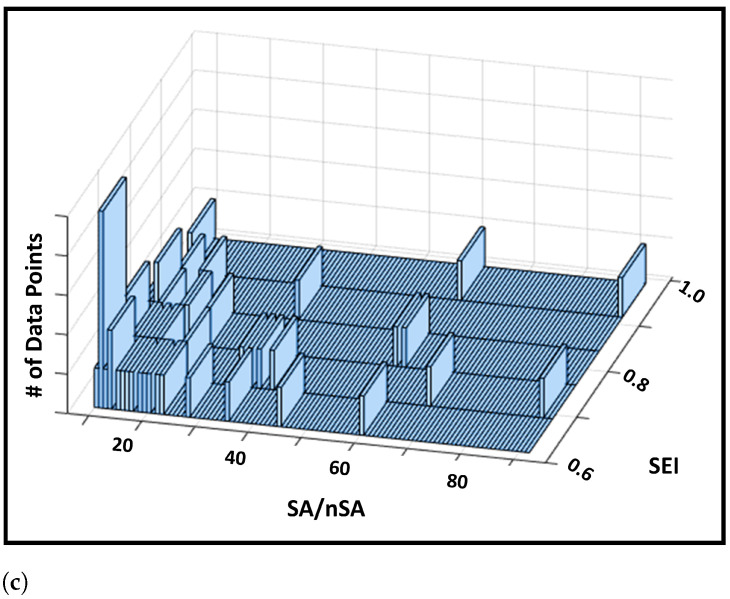
This figure displays the linear trend between SEI and patient encounter ratio.SEI results from 1000 patients. SA = Encounters labeled 1; nSA = Encounters labeled 0. Higher the SA/nSA ratio, higher the substance dependence, and higher the SEI. (**a**) SEI Range: 0 < SEI < 0.6; SA/nSA Ratio Range: 0 ≤ (SA/nSA) < 1. (**b**) SEI Range: 0.4 < SEI < 0.8; SA/nSA Ratio Range: 1 ≤ (SA/nSA) < 10. (**c**) SEI Range: 0.6 < SEI ≤ 1; SA/nSA Ratio Range: 10 ≤ (SA/nSA) < 90.

In this section we validate whether using an aggregation function, such as *mean*, is appropriate for defining SEI. To do so, we calculate the ratio of substance abuse to non-substance abuse encounters for each patient, and denote it as SA/nSA (see Figure 4). We expect to find that higher values of the SA/nSA ratio corresponding to higher values of SEI. Figure 4, which is a 3D plot of SEI versus SA/nSA ratio versus the total number of data points in each group, shows the expected trend, even though the trend is step-wise linear. Figure 4a–c are subplots of the trend concept, separated by the range of SEI and SA/nSA ratio. The respective ranges for the subplots are given in the respective sub-captions.

#### 3.3.2. Patient Level Information Encapsulation

In Section 3.2, we validated the LR model at encounter level, because the purpose of the LR model was to convert the hard labels and assign soft labels to each encounter. SEI, however, is a patient level metric (obtained from the aggregation of soft labels.) Thus, in this section, we evaluate SEI as a dependent variable metric produced by a patient. If we can achieve good correlation between SEI and patient attributes that are related to substance abuse, but which were never used in the SEI development process, then we can state with stronger confidence that SEI encapsulates substance abuse information about each patient.

In order to obtain these “related” but “unused” attributes, we divided the EMR tables into two sets (Figure 1): one, for developing SEI (e.g., the Diagnosis, Laboratory, Procedure and Medication tables), and another for validating SEI (the Encounters and Clinical Events tables). Table 1 and Table 2 also show that we used data only from the Diagnosis, Laboratory, Procedure and Medication tables to train the LR model described by Equation (6). The logic here is that even though the Encounters and Clinical Events tables contain different attributes than the Diagnosis, Laboratory, Procedure and Medication tables, some of the attributes between the two sets of tables should have high enoguh correlation. Thus, if SEI truly embodies the trends in the EMR data, then we should be able to regress the SEI values for the individual patients (not encounters), using the attributes from the Encounters and Clinical Events. Thus, using SEI as the response variable and the attributes from the Encounters and Clinical Events as the independent variables, we apply the following regression models to the data subset: (i) the Decision Tree regression, and (ii) the Gaussian Process regression.

(i) Regression Tree: Similar to classification with Decision Trees [24], the Decision Tree for regression splits the attributes into mutually exclusive intervals to create step-wise linear decision boundaries. However, unlike classification trees (which uses entropy and information gain for branching), the regression tree splits the attributes using the *mean square error* (MSE), and thus, the leaf nodes of the regression tree consists of the attribute’s value at the node, the number of samples at the leaf node (e.g., the leaf’s size), and the MSE between the attribute’s node value and the attribute’s samples’ values.
(ii) Gaussian Process Regression: The Gaussian Process [25] assumes an infinite set of variables, where any finite collection of variables follow a joint multivariate Gaussian joint distribution. Thus, this regression model predicts the response variable by optimizing the covariance function of this distribution, while setting the mean to 0.

**Figure 5:**
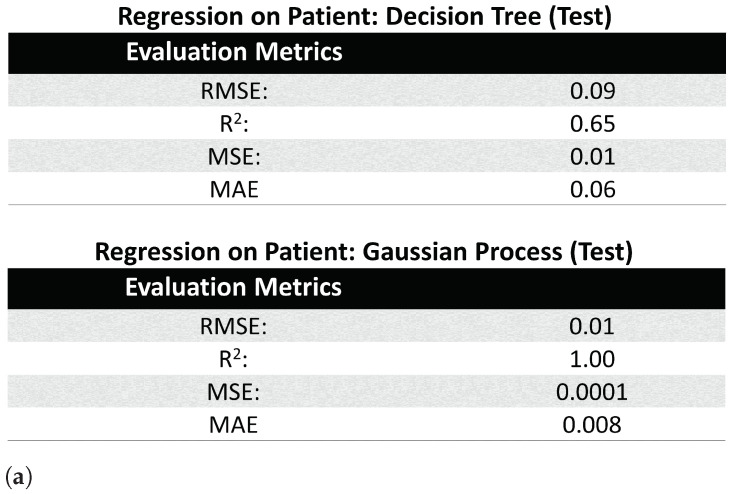

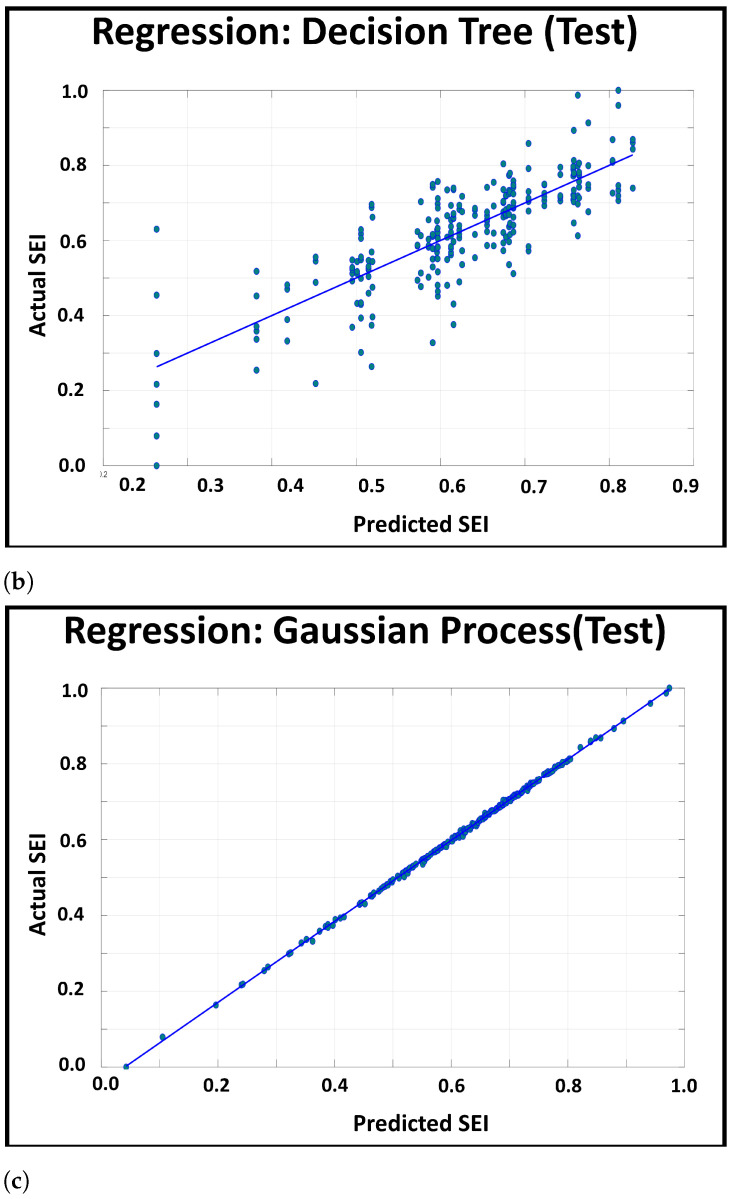
Regression results displaying how much of substance abuse information was encapsulated by SEI, even though only four of the six EMR tables were used for its definition. (**a**) Value of evaluation metrics often used in regression. (**b**) Decision Tree Regression: Actual vs. Predicted SEI. (**c**) Gaussian Process Regression: Actual vs. Predicted SEI.

For implementation, we used Matlab’s in-built Fine Decision Tree regression model, allowing the minimum leaf size to be 4. The results for Decision Tree are shown in Figure 5a,b. For the Gaussian Process regression model, we once again used Matlab’s in-built function, with a constant basis function and an exponential kernel, where parameters such as the kernel scale, were set to be automatically computed by the computer. The results for Gaussian Process are shown in Figure 5a,c. The models were trained on 80% patient data, and tested on the remaining 20% of the patient data. We used four evaluation metrics, 3 error (Root Mean Square Error (RMSE), Mean Square Error (MSE), and Mean Absolute Error (MAE)) and 1 correlation (R- squared ($R^{2}$) ) metrics to validate the regression analysis. The formula for the evaluation metrics used in Figure 5a is given below:

(10) $$MSE=\frac{1}{n}\sum_{i=1}^{N}{\left(y_{i}-\hat{y_{i}}\right)}^{2}$$

(11) $$RMSE=\sqrt{\frac{1}{n}\sum_{i=1}^{N}{\left(y_{i}-\hat{y_{i}}\right)}^{2}}$$

(12) $$MAE=\frac{1}{n}\sum_{i=1}^{N}\left|y_{i}-\hat{y_{i}}\right|$$

(13) $$R^{2}=\frac{n\sum_{i=1}^{N}x_{i}y_{i}-\sum_{i=1}^{N}x_{i}\sum_{i=1}^{N}y_{i}}{\sqrt{\left[n\sum_{i=1}^{N}{x_{i}}^{2}-{\left(\sum_{i=1}^{N}x_{i}\right)}^{2}\right]\left[n\sum_{i=1}^{N}{y_{i}}^{2}-{\left(\sum_{i=1}^{N}y_{i}\right)}^{2}\right]}}$$

From Figure 5, we find that the error metrics for the both regression models are quite low, with the Decision Tree and Gaussian Process models reaching a $R^{2}$ value of 0.65 and 1.00 respectively. Plots in Figure 5b,c display a linear trend between actual and predicted SEI, even though the intercept is not zero for either models, showing that SEI is highly correlated with the attributes in the Encounter and Clinical Events tables.

## 4. Discussion

To the best of our knowledge, this is the first step to define and develop an objective metric that measures the tendency of a patient for substance abuse that may used in conjunction with psychometric scores. And this paper explores the feasibility of defining SEI from EMR patient database. The purpose of SEI is to combine the influence of different factors that leads to substance abuse into one single scalar measure. Factors that affect substance abuse can be biological, psychological, physiological, environmental or any combination of multiple factors. Since SEI is developed from EMR data, we expect SEI to have ability to encapsulate the biological and (to a certain extent) the environmental and physiological influences that can lead to substance abuse.

In this paper, we were able to test and validate one of the conducive developmental methods of developing SEI using EMR dataset and data mining algorithms. Since the worst possible value for AUROC is 0.5 representing a model with no discriminatory ability, and the best possible value is 1.0 [26], we conclude that a AUROC high value of 0.95 (or 95%) is quite high Figure 2a. Similar results are reflected with other evaluation metrics (such as accuracy, specificity and sensitivity) in Figure 2b, where a random classifier with no discriminatory ability is expected to have an accuracy of 50% [27]. Thus, once again we conclude that the LR model has good performance on the EMR dataset, which provides good evidence to trust the probabilities produced by the model. In addition, we also evaluated the results at patient level through visual validation (Figure 4) and other regression models (Figure 5) on a different but related subset of the original dataset (Figure 1). Figure 4 shows a linear trend between SEI (from the LR model) and the ratio of expert labeled substance encounters to non-substance encounters as we expected (Section 3.3.1). On the other hand, in Section 3.3.2, Figure 5 shows that even though the Encounter and Clinical Events tables were not used by the LR model, the output of LR model (e.g., SEI values) correlate well with the pattern present in these two tables, which include the same patients. Thus, based on the results presented in Section 3, we were able to show that the proposed SEI developed using logistic regression and Equation (6) is able to encapsulate substance abuse related information, and can be improved further. The results are therefore strong and promising.

In par with Health Insurance Portability and Accountability Act (HIPAA) regulations, we cannot share any patient level information/figures that can cause a breach in the privacy of a patient; and hence we presented no traceable information in Section 3. As such, we want to take this opportunity to discuss the interaction between SEI and the medication prescribed to each patient as an overall observation, without involving specific patient data. These interactions are, to say the least, very complex and varies from patient to patient. Use of some medications such as opiates, clonazepam, and nicotine, pushes the SEI values towards the higher end and usually above the th value of 0.56. Some drugs, such as ondanestron, which is not known to have an addictive ingredient, also played an important role in pushing up the SEI value. This can be due to two reasons: one, ondanestron is used to treat addiction and thus, signal substance abuse cases, and/or two, fear and anxiety can make it difficult to give up using ondanestron.

There were two other classes of drugs that are not generally known to be addictive, but had high SEI values; one of these classes involved anti-psychotic drugs that have a direct effect on the central nervous system (e.g., prochlorperazine). Ref. [28] found effective (but not necessarily causal) correlation among substance abuse and schizophrenic patients. The other class was a bit surprising and involved insulin related medication and/or vitamin B related medications (especially, when combined with medications like methadone or methocarbamol). While the relationship between substance abuse and insulin/vitamin intake is not clear, ref. [29] found that insulin pathway have an effect on the central nervous system, which in return could make a person more inclined towards “craving”, and [30], a case study, warns against the unexpected abuse of Vitamin D. Thus, based on these observations and based on the results presented in Section 3, we conclude that the proposed SEI developmental route is promising and warrants further investigation and work for deeper insights.

**Future Work.** As such, our next steps towards developing SEI based on some of the inferences and questions from this work are as follows:

First, since we use logistic regression for obtaining soft labels in this paper, SEI is restricted to lie between 0 and 1. The only reason we favored the range of SEI to lie between 0 and 1, is because a bounded variable is easier to study; however, there is no practical or physical concerns that dictates that SEI needs to be bounded. As our future work, we expect to conduct a comparison study between a bounded SEI variable and an unbounded SEI variables. Similarly, even though logistic regression seems to be the most appropriate model for obtaining the soft labels, analyzing the expression in Equation (6), we realize that obtaining the soft labels may boil down to the obtaining the optimized coefficients ($\beta_{i}^{*}$ in Equation (6)). Thus, other machine learning models, which are capable of binary classification through optimization of weights (e.g., finding $\beta_{i}^{*}$ equivalents) for given attributes, can be used as well. Experimenting with different machine learning models, which can handle non-linear patterns, may also allow us to obtain better sensitivity and specificity [24,25,31,32,33].
Second, the interaction that SEI has with medication and other attributes in the EMR dataset might be worth deeper understanding. Developing a system that can trace the interactions in a complex system, or using some of the existing technology [34], may shed new light into the factors and their influence on individuals for substance abuse.
Third, note that SEI does not need to be a function of just an EMR dataset. Using computational techniques of multi-modal dataset analysis, SEI can be developed from a combined dataset of EMR data and survey questionnaires to incorporate the psychological aspect of substance abuse.

**Limitations.** Finally, the evaluation of SEI was rather challenging at this stage of development as the ultimate evaluation would involve conducting clinical trials (since there is no benchmark for the index). Instead in this paper, we enlisted the aid of our medical collaborator who acted as the medical expert for handling the data, and had to rely on computational evaluation techniques (described in Section 3) for understanding the appropriateness of the proposed SEI. Another limitation of the proposed workflow is that the current definition of SEI does not account for temporal changes e.g., the current model does not differentiate a person at the final stage of substance abuse treatment from a person at initial stage of treatment. A third limitation might be the quality of EMR datasets in general, but EMR databases are still the best source for obtaining objective, biological, and expert annotated data.

## 5. Conclusions

In this paper we test the feasibility of developing a substance effect index (SEI), with the goal of ultimately developing a standardized, objective metric that can successfully measure the patient level tendency to abuse particular substances. Due to objective nature of biological data, we define SEI to be a function of EMR dataset, and used logistic regression to find a closed form mathematical expression for SEI by re-calibrating hard coded labels obtained from EMR diagnostic codes. Validations of both the LR model and SEI are promising, and show evidence of SEI encapsulating interactions between various attributes, including medication, that are related to substance abuse. Thus, we conclude that pursuing further development of SEI to worthwhile. Based on the results obtained in this paper, we expect to develop a better SEI model by using other machine learning models, which can overcome some of the limitations of our current model.

## Figures and Tables

**Table 1:** An overview of the tables/relations, along with few of their attributes, that contain the patient data and compose the EMR dataset.The relations used in the development of SEI are shown in bold (see Section 3 for more details).

Table Names	Relevant Attributes
Encounters	Encounter Id, Patient Id, Age, Weight, Geography, Race, Gender, Admission type, Health Insurance, Timestamp
**Procedures**	Procedure description, Procedure Priority, Timestamp
**Diagnosis**	Diagnosis Type, Diagnosis Code, Diagnosis Description, Timestamp
Clinical Events	Event Descriptions, Results, Range, Timestamp
**Laboratory**	Blood Test, Urine Test, Other fluid test, Drug Screening, Results, Range, Timestamp
**Medication**	Generic Name, Specific Name, Dosage, Timestamp

**Table 2:** The tables/relations and the corresponding attributes from the EMR dataset that were used for obtaining soft labels in the definition of SEI (see Section 2.4).

Tables Used in SEI Definition	Selected Attributes
**Diagnosis**	EncounterId, DiagnosisId, DiagnosisType, DiagnosisCode, DiagnosisDescription, ConditionCategory, DiagnosisPriority, DiagnosisTypeId, DiagnosisTypeDisplay, PresentOnAdmitId
**Laboratory**	EncounterId, DetailLabProcedureId, LabProcedureName, LabProcedureGroup, LabSuperGroup, ResultIndicatorId, ResultIndicatorDesc, Accession, NumericResult, ResultUnitsId, UnitDesc, LabOrderedDtTm, LabDrawnDtTm, LabReceivedDtTm, LabPerformedDtTm, LabVerifiedDtTm, LabCompletedDtTm
**Procedure**	EncounterId, ProcedureId, ProcedureType, ProcedureCode, ProcedureDescription, ProcedurePriority, ProcedureDtTm
**Medication**	EncounterId, MedicationId, NdcCode, BrandName, GenericName, ProductStrengthDescription, RouteDescription, DoseFormDescription, FrequencyId, FrequencyDesc, TotalDispensedDoses, DoseQuantity, InitialDoseQuantity, DoseUnitsId, DoseUnitsDesc, OrderStrength, OrderStrengthUnitsId, OrderStrengthUnitsDisp, MedEnteredDtTm, MedStartedDtTm, MedStoppedDtTm, MedDiscontinuedDtTm

## Data Availability

The dataset used here is private and regulated by HIPAA and OSU CHSI. Usage requires special permission from OSU CHSI.

## References

[1] Koob G.F., Volkow N.D.T. (2010). Neurocircuitry of addiction. Neuropsychopharmacology.

[2] Tsuang M.T., Lyons M.J., Meyer J.M., Doyle T., Eisen S.A., Goldberg J., True W., Lin N., Toomey R., Eaves L. (1998). Co-occurrence of abuse of different drugs in men: The role of drug- specific and shared vulnerabilities. Arch. Gen. Psychiatry.

[3] Kendler K.S., Myers J., Prescott C.A. (2007). Specificity of genetic and environmental risk factors for symptoms of cannabis, cocaine, alcohol, caffeine, and nicotine dependence. Arch. Gen. Psychiatry.

[4] Dong X., Deng J., Rashidian S., Abell-Hart K., Hou W., Rosenthal R.N., Saltz M., Saltz J.H., Wang F. (2021). Identifying risk of opioid use disorder for patients taking opioid medications with deep learning. J. Am. Med. Inform. Assoc..

[5] Shahriar A., Faisal F., Mahmud S.U., Chakrabarti A., Rabiul Alam M.G. A Machine Learning Approach to Predict Vulnerability to Drug Addiction. Proceedings of the 22nd International Conference on Computer and Information Technology (ICCIT).

[6] Conway K.P., Levy J., Vanyukov M., Chandler R., Rutter J., Swan G.E., Neale M. (2010). Measuring addiction propensity and severity: The need for a new instrument. Drug Alcohol Depend..

[7] Skinner H.A., Allen B.A. (1982). Alcohol dependence syndrome: Measurement and validation. J. Abnorm. Psychol..

[8] Stockwell T., Hodgson R., Edwards G., Taylor C., Rankin H. (1979). The Development of a Questionnaire to Measure Severity of Alcohol Dependence. Br. J. Addict. Alcohol Other Drugs.

[9] Tarter R.E., Laird S.B., Kabene M., Bukstein O., Kaminer Y. (1990). Drug abuse severity in adolescents is associated with magnitude of deviation in temperament traits. Br. J. Addict..

[10] McLellan A.T., Luborsky L., Woody G.E., O’Brien C.P. (1980). An improved diagnostic evaluation instrument for substance abuse patients: The addiction severity index. J. Nerv. Ment. Dis..

[11] Dennis M.L., Kaminer Y. (2006). Introduction to special issue on advances in the assessment and treatment of adolescent substance use disorders. Am. J. Addict..

[12] Kirisci L., Mezzich A., Tarter R. (1995). Norms and sensitivity of the adolescent version of the drug use screening inventory. Addict. Behav..

[13] Ovalle A., Goldstein O., Kachuee M., Wu E.S.C., Hong C., Holloway I.W., Sarrafzadeh M. (2021). Leveraging Social Media Activity and Machine Learning for HIV and Substance Abuse Risk Assessment: Development and Validation Study. J. Med. Internet Res..

[14] Barenholtz E., Fitzgerald N.D., Hahn W.E. (2020). Machine-learning approaches to substance-abuse research: Emerging trends and their implications. Curr. Opin. Psychiatry.

[15] Garrouste-Orgeas M., Troché G., Azoulay E., Caubel A., de Lassence A., Cheval C., Montesino L., Thuong M., Vincent F., Cohen Y. (2004). Body mass index. Intensive Care Med..

[16] Trung T.Q., Le H.S., Dang T.M.L., Ju S., Park S.Y., Lee N.-E. (2018). Freestanding, Fiber-Based, Wearable Temperature Sensor with Tunable Thermal Index for Healthcare Monitoring. Adv. Healthcare Mater..

[17] Humphreys J.S. (1998). Delimiting ‘Rural’: Implications of an Agreed ‘Rurality’ Index for Healthcare Planning and Resource Allocation. Aust. J. Rural. Health.

[18] Cheong K.H., Tang K.J.W., Zhao X., Koh J.E.W., Faust O., Gururajan R., Ciaccio E.J., Rajinikanth V., RajendraAcharya U. (2021). An automated skin melanoma detection system with melanoma-index based on entropy features. Biocybern. Biomed. Eng..

[19] Rios A., Kavuluru R. (2019). Neural transfer learning for assigning diagnosis codes to EMRs. Artif. Intell. Med..

[20] Yang B., Dai G., Yang Y., Tang D., Li Q., Lin D., Zheng J., Cai Y. (2018). Automatic Text Classification for Label Imputation of Medical Diagnosis Notes Based on Random Forest. Health Inf. Sci..

[21] Wu L.-T., Ringwalt C.L., Williams C.E. (2003). Use of Substance Abuse Treatment Services by Persons With Mental Health and Substance Use Problems. Psychiatr. Serv..

[22] Kleinbaum D.G., Klein M. (2002). Logistic Regression.

[23] Suthar B., Patel H., Goswami A. (2012). A survey: Classification of imputation methods in data mining. Int. J. Emerg. Technol. Adv. Eng..

[24] Suthaharan S. (2016). Decision Tree Learning. Machine Learning Models and Algorithms for Big Data Classification. Integrated Series in Information Systems.

[25] Rasmussen C.E., Bousquet O., von Luxburg U., Rätsch G. (2004). Gaussian Processes in Machine Learning. Advanced Lectures on Machine Learning.

[26] Hoo Z.H., Candlish J., Teare D. (2017). What is an ROC curve?. Emerg. Med. J..

[27] Zhu W., Zeng N., Wang N. (2010). Sensitivity, specificity, accuracy, associated confidence interval and ROC analysis with practical SAS implementations. NESUG Proc. Health Care Life Sci..

[28] Cuffel B.J., Heithoff K.A., Lawson W. (1993). Correlates of Patterns of Substance Abuse Among Patients With Schizophrenia. Psychiatr. Serv..

[29] Daws L.C., Avison M.J., Robertson S.D., Niswender K.D., Galli A., Saunders C. (2011). Insulin signaling and addiction. Neuropharmacology.

[30] Bharti K., Singh B.Y.P., Sanjay K. (2012). Addiction to vitamin D: Unusual, unexpected substance abuse. J. Acad. Med Sci..

[31] Steele V.R., Maurer J.M., Arbabshirani M.R., Claus E.D., Fink B.C., Rao V., Calhoun V.D., Kiehl K.A. (2018). Machine Learning of Functional Magnetic Resonance Imaging Network Connectivity Predicts Substance Abuse Treatment Completion. Biol. Psychiatry Cogn. Neurosci. Neuroimaging.

[32] Acion L., Kelmansky D., van der Laan M., Sahker E., Jones D., Arndt S. (2017). Use of a machine learning framework to predict substance use disorder treatment success. PLoS ONE.

[33] Nath P., Kilam S., Swetapadma A. A machine learning approach to predict volatile substance abuse for drug risk analysis. Proceedings of the Third International Conference on Research in Computational Intelligence and Communication Networks (ICRCICN).

[34] Lee E., Braines D., Stiffler M., Hudler A.A., Harborne D. Developing the sensitivity of LIME for better machine learning explanation. Proceedings of the Artificial Intelligence and Machine Learning for Multi-Domain Operations Applications, SPIE.

